# Entomopathogenic Fungus as a Biological Control for an Important Vector of Livestock Disease: The *Culicoides* Biting Midge

**DOI:** 10.1371/journal.pone.0016108

**Published:** 2011-01-10

**Authors:** Minshad Ali Ansari, Edward C. Pope, Simon Carpenter, Ernst-Jan Scholte, Tariq M. Butt

**Affiliations:** 1 Pure and Applied Ecology, Swansea University, Swansea, United Kingdom; 2 Vector-Borne Disease Programme, Institute for Animal Health, Pirbright, United Kingdom; 3 National Centre for Monitoring of Vectors, Ministry of Agriculture, Nature and Food Quality, Wageningen, The Netherlands; Universidade Federal de Minas Gerais, Brazil

## Abstract

**Background:**

The recent outbreak of bluetongue virus in northern Europe has led to an urgent need to identify control measures for the *Culicoides* (Diptera: Ceratopogonidae) biting midges that transmit it. Following successful use of the entomopathogenic fungus *Metarhizium anisopliae* against larval stages of biting midge *Culicoides nubeculosus* Meigen, we investigated the efficacy of this strain and other fungi (*Beauveria bassiana*, *Isaria fumosorosea* and *Lecanicillium longisporum*) as biocontrol agents against adult *C. nubeculosus* in laboratory and greenhouse studies.

**Methodology/Findings:**

Exposure of midges to ‘dry’ conidia of all fungal isolates caused significant reductions in survival compared to untreated controls. *Metarhizium anisopliae* strain V275 was the most virulent, causing a significantly decrease in midge survival compared to all other fungal strains tested. The LT_50_ value for strain V275 was 1.42 days compared to 2.21–3.22 days for the other isolates. The virulence of this strain was then further evaluated by exposing *C. nubeculosus* to varying doses (10^8^–10^11^ conidia m^−2^) using different substrates (horse manure, damp peat, leaf litter) as a resting site. All exposed adults were found to be infected with the strain V275 four days after exposure. A further study exposed *C. nubeculosus* adults to ‘dry’ conidia and ‘wet’ conidia (conidia suspended in 0.03% aq. Tween 80) of strain V275 applied to damp peat and leaf litter in cages within a greenhouse. ‘Dry’ conidia were more effective than ‘wet’ conidia, causing 100% mortality after 5 days.

**Conclusion/Significance:**

This is the first study to demonstrate that entomopathogenic fungi are potential biocontrol agents against adult *Culicoides*, through the application of ‘dry’ conidia on surfaces (e.g., manure, leaf litter, livestock) where the midges tend to rest. Subsequent conidial transmission between males and females may cause an increased level of fungi-induced mortality in midges thus reducing the incidence of disease.

## Introduction

*Culicoides* biting midges are widely distributed throughout the world and are vectors of internationally important livestock viruses, including bluetongue virus (BTV), African horse sickness virus (AHSV), Akabane virus and Epizootic haemorrhagic disease virus (EHDV) [1]. Bluetongue disease (BT) has gained considerable notoriety in recent years because of an unprecedented globalisation and climate change-mediated expansion of its range in Europe, resulting in BTV reaching areas with no historical record of the disease [2], [3]. The economic impact of outbreaks of BTV in these areas has been considerable as a result of both indirect costs (e.g. the restrictions placed on movement of infected ruminants) and direct losses from disease in both sheep and cattle. In addition, whilst vaccination campaigns across northern Europe eventually controlled outbreaks in this region, it is noteworthy that it took approximately eighteen months from the initial incursion in 2006 to the deployment of vaccine in the field [4]. During this lag period, attempts to control the spread of BTV were limited to the restriction of animal movement and the application of methods to control *Culicoides* midges (primarily through the use of pour-on pyrethroid insecticides to vulnerable stocks).

BTV is an arbovirus and therefore depends almost entirely on the occurrence of farm-associated populations of competent *Culicoides* biting midges for transmission to its ruminant hosts. As a period of extrinsic replication is required within these vectors, control measures directed at adults have the potential to reduce the spread of midge-transmitted diseases through shortening or interrupting their lifespan. Indeed, epidemiological transmission models of vector-borne diseases show that the adult lifespan is the single most important factor affecting risk of transmission [5]. At present, the majority of approaches to control populations of biting midges are based upon the application of insecticides (primarily synthetic pyrethroids) which in northern Europe are most commonly applied to livestock, although systematic testing of compounds to date has demonstrated equivocal results [6]. Wide scale larvicidal or adulticidal use of these compounds against *Culicoides* has not been considered sustainable because of the paucity of knowledge surrounding larval habitats and adult resting places, combined with increasing restrictions within the EU on untargeted use of pyrethroid insecticides. An alternative insecticide, Ivermectin, is effective in killing *Culicoides* species when applied intradermally or subcutaneously and also toxic to midge larvae when excreted in faeces (a potential breeding site) but has also been shown to be harmful to beneficial insects such as dung beetles [7]. Farmers are therefore caught between the need to control populations of biting midges and the diminishing number of chemical insecticides as they are withdrawn because of their perceived risk to humans and the environment [4].

There is therefore an increasing interest in alternative and integrated vector control methods, including biocontrol. Entomopathogenic fungi, *Metarhizium anisopliae*, *Beauveria bassiana* (Balsamo) Vuillemin, *Lecanicillium* ( =  *Verticillium*) *lecanii* (Zimmermann), and *Isaria* (* = Paecilomyces*) *fumosorosea* Wize are already commercially available [8] and successfully used to control both agricultural insect pests [9], [10] and insect vector species able to transmit diseases to humans [11], [12]. Whilst a few attempts have been made to control *Culicoides* larvae with the fungus *Culicinomyces clavisporus* [13], [14] and recent studies using *M. anisopliae* against *C. nubeculosus* larvae have given promising results [15], there is still an increasing need to investigate the potential of entomopathogenic fungi against adult midges, the life stage potentially more easily targeted than larvae.

To date, there are no reports on the efficacy of fungus against adult midges. In this study we investigated the effectiveness of four different potential fungal biocontrol pathogens, *M. anisopliae*, *B. bassiana*, *I. fumosorosea* and *L. longisporum* against adult *C. nubeculosus*. The midge species was chosen in this study because it is endemic to the UK and one of the few species that can be cultured in a laboratory. Adult midges were exposed to different substrates treated with ‘dry’ conidia and conidia suspended in 0.03% aq. Tween 80 (hereafter referred as ‘wet’ conidia) of fungal strains. This is the first study of this type to use different substrates (peat, leaf litter, horse manure) as a representative resting site for *Culicoides* midges and therefore allows a more accurate estimation of the efficacy of fungus in the field.

## Materials and Methods

### Biting midges

A mixed population of male and female adult *C. nubeculosus* was provided by the Institute for Animal Health, Pirbright, Surrey, UK from an existing colony. These adults were maintained at a constant 20°C and 70% r.h. and provided with small balls of cotton wool soaked in a 10% sucrose solution, both before and during transportation to the laboratory. Midges were used in the experiments when 3–4 days old.

### Fungus

Five commercially available fungal strains were used: *M. anisopliae* V275 ( =  BIPESCO 5; F52, isolated from *Cydia pomonella*, Austria); *B. bassiana* BotaniGard® (provided by Mycotech Corporation, USA, isolated from a *Diabrotica* spp., Coleoptera, USA); *I. fumosorosea* PFR 97 (provided by Certis Biological, UK, isolated from *Phenacoccus* sp., USA); *I. fumosorosea* strain CLO 55 (isolated from soil; *Galleria* bait, Belgium) and *Lecanicillium* (* = Verticillium*) *longisporum* (Vertalec® strain; provided by Koppert Biological Systems, The Netherlands). Fungal strains were passed through *Galleria mellonella* larvae to ensure the cultures were not attenuated and re-isolated on oatmeal dodine agar medium. Single spore colonies were transferred to Sabouraud Dextrose Agar (SDA) and incubated at 25±1°C for 15 days. Conidia obtained from the first subculture were used for mass production of inoculum.

Aerial conidia of fungus were produced on broken Basmati rice (East End Foods plc, West Midlands, UK) as previously described [16] with slight modification. After harvest, conidia were dried at room temperature until moisture content was <5%. To determine the number of conidia g^−1^ dry powder, 0.1 g suspended in 100 ml of 0.03% (vol/vol) aqueous Tween-80 (Fisher Scientific, Leicestershire, UK) was counted using a haemocytometer (Weber Scientific, Teddington, UK) under a light microscope (400× magnification). Conidial viability was assessed using the plate count technique on SDA [17] and viability was >95% for all strains. Prior to use, ‘dry’ conidia were stored in air tight plastic containers in the dark at 4°C.

### Fungal susceptibility test

**Figure 1:**
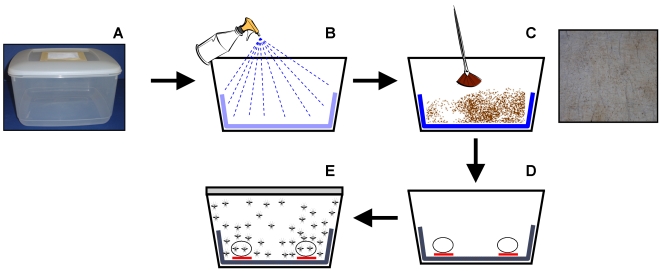
Experimental procedure. Protocol used to contaminate adult *Culicoides nubeculosus* biting midges with ‘dry’ conida of entomopathogenic fungi. (A) Experimental vessels were white opaque plastic containers (25×25 cm; 15 cm in depth; surface area 625 cm^2^) with a ventilation hole (10×10 cm) cut into the lids and covered with nylon gauze (64 µm pore size). (B) A double layer of tissue tissue paper (36.5 cm length; 25 cm width; surface area 917.5 cm^2^) was placed in each container so that it covered the bottom and halfway up each side. This tissue paper was then moistened using a hand-held sprayer. (C) ‘Dry’ conidia of entomopathogenic fungi were uniformly dusted on the tissue paper using a paintbrush. Inset shows a photograph of moist tissue paper dusted with *Metarhizium anisopliae* (10^11^ conidia m^−2^) to illustrate distribution of conidia. (D) Two cotton wool balls soaked in 10% glucose/water (w/v) solution (placed on plastic trays to prevent the solution soaking into the tissue paper) were provided as a food source. (E) Approximately 40 adult male and female midges were released into the containers and midge survival monitored daily for 6 days.

These experiments were designed to evaluate the virulence of different fungal species/strains against adult *C. nubeculosus*. Assays were conducted in white opaque plastic containers (25×25 cm; 15 cm in depth; surface area 625 cm^2^) (Wilkinsons Ltd, Swansea, UK). One ventilation hole (10×10 cm) was made in each lid and covered with nylon gauze (64 µm pore size). A double layer of moist tissue paper (Kruger Ltd, UK) was placed in each container so that it covered the bottom and halfway up each side. This tissue paper was then dusted with ‘dry’ conidia of each fungal species/strain at the rate of 10^11^ m^−2^ using a small paintbrush (Fig. 1). Two cotton wool balls soaked in 10% glucose/water (w/v) solution (placed in plastic trays to prevent the solution soaking into the tissue paper) were provided as a food source.

For each replicate, approximately 40 adult male and female midges were released into each container. Midges were therefore continually exposed to conidia through tarsal contact or on the head and thorax region for the duration of the study. The tissue paper remained in the container until the end of the test (a minimum of 6 days). Control midges were treated in the same way but in the absence of conidia. Containers were kept in a constant temperature room (20±1°C, 80–90% r.h., and L16: D8). Midge survival was monitored daily for 6 days. Dead midges were collected individually from each container, dipped in 70% ethanol, and incubated on moist tissue paper in Petri dishes (25±1°C for 3–5 days) after which they were examined using a light microscope at magnification 40× for evidence of fungal sporulation. Each treatment was replicated four times and the whole experiment was conducted twice.

### Dose-response experiments

The fungal susceptibility test identified *M. anisopliae* V275 as highly virulent (Table 1) and so it was selected for further investigation. These experiments were conducted as already described except different doses (10^8^, 10^9^, 10^10^ and 10^11^ ‘dry’ conidia m^−2^) were dusted on the surface of separate substrates (tissue paper, peat or horse manure). A single layer of moist tissue paper was used as described earlier or moist peat (0.5 L; Bord Na Mona, Newbridge, Ireland) or horse manure (0.5 L; obtained from local livestock) were evenly spread on the bottom of the container before application of conidia. A fourth test used the same doses of ‘wet’ conidia sprayed onto tissue paper with a hand held sprayer (Minijet, SATA, Germany). For each replicate, approximately 40 male and female adult midges were introduced into each container 4 hrs after fungal application. Control midges were treated in the same way but in the absence of conidia. Preliminary studies showed that there were no differences in midge survival among different substrates, so we included only one control (moist tissue paper) for data analysis. Midge survival was monitored daily for 6 days. Dead midges were collected individually as mentioned above and examined using a light microscope at magnification ×40. All doses were replicated four times per experiment and each experiment was conducted twice.

**Table 1:** Mean lethal time.

Fungus species/strains	LT_50_(days)*	LT_90_(days)*
*Metarhizium anisopliae* V275	1.42 (1.35–1.50)	3.26 (3.08–3.44)
*Beauveria bassiana* BG	2.21 (2.09–2.34)	4.69 (4.40–5.09)
*Isaria fumosorosea* CLO55	2.74 (2.6–2.89)	5.76 (5.31– nc)
*Lecanicillium longisporum*	2.93 (2.77–3.09)	5.91 (5.38–nc)
*Isaria fumosorosea* PFR 97	3.22 (3.01–3.44)	5.99 (5.46–nc)

Adult midges were exposed to tissue paper treated with ‘dry’ conidia of entomopathogenic fungus at dose of 10^11^ conidia m^−2^ at 20±1°C. Controls were not exposed to any fungus (‘0’ doses). Dead midges were collected daily from each container for 6 days and kept at 25°C for sporulation.

* =  Mean lethal time (time taken in days to kill 50 and 90% of midges) estimated (four replicates/dose; approximately 40 adult males and females/replicate). 95% Confidence intervals in parentheses.

nc  =  not calculated (insufficient data).

### Substrate and formulation experiments

Greenhouse experiments using adult midges and different substrates and formulations were conducted to evaluate the efficacy of most virulent strain, V275, in conditions more representative of those in the wild. Experiments were conducted in cages (75×75×75 cm) covered with nylon gauze (64 µm pore size). A plastic tray (70×70×4.5 cm) filled (*ca.* 4 cm depth) with either moist peat or leaf litter (predominantly beech *Fagus sylvatica*) was placed inside each cage. Two experiments used ‘dry’ V275 conidia dusted uniformly on each substrate using a paintbrush to give final dose of 2.5×10^9^ conidia m^−2^ substrates. Two later experiments used the same dose of conidia suspended in 0.5 L water containing 0.03% Tween 80 (‘wet’ conidia) and uniformly sprayed over the surface of the peat and leaf litter using a hand held sprayer operating at a constant pressure of 2 bars. The nozzle of the spray gun was held 50 cm away from the application surface and adults were introduced into the cage 4 h after conidial application. The conidial dose was verified after spraying by sampling each substrate using a squire block (three 3×3×2 cm samples). These samples (ca. 20 ml) were subsequently suspended in 500 ml Erlenmeyer flasks containing 100 ml 0.03% Tween and placed on a rotary shaker (Gallenkamp, UK) at 120 rpm for 10 min. Conidia were separated from substrate materials by filtration of the suspension through a filter cloth (Calbiochem, Darmstadt, Germany) and the number of conidia determined using a hemocytometer. The same volume of each substrate was then returned to the sampling sites.

Approximately 200 male and female adult midges were released into each cage and 4 small cotton balls soaked in 10% glucose were placed in each corner as a food source. Control cages experienced identical conditions to the treatment cages but were not treated with fungus. Survival was assessed daily for 6 days by deploying two sticky traps (AgriSense, Pontypridd, UK) in each cage at 10:00 h for 2 hrs. These sticky traps capture midges in flight and a deployment time of 2 hrs was sufficient to capture all surviving midges within a cage, allowing percent survival to be calculated. This meant that each cage could be sampled once only and therefore 75 cages in total were used (three replicates of five treatments sampled daily for five days) per experiment and the entire experiment was conducted twice. To investigate infection of surviving midges, midges caught on the sticky traps were placed on moist filter paper in Petri dishes, sealed with Parafilm and incubated at 25±1°C for 3–5 days. After this incubation period, midges were examined for evidence of fungal sporulation (i.e. emerging hyphae) using a dissection microscope and the number infected recorded. The air temperature in the greenhouse ranged from 20 to 22°C during the experiments and the corresponding temperature of the substrates (peat or leaf litter) at 3 cm depth ranged from 18–20°C.

### Data analysis

Differences in midge survival between fungus-infected and control groups were analysed using the Kaplan-Meier method to plot cumulative survival functions by treatment with pairwise comparison conducted using the log-rank test [18] (SPSS v. 16). Mean lethal time (LT_50_) and (LT_90_) values were calculated by fitting the data to the Gompertz distribution model using GraphPad Prism v. 5. Mean lethal dose (LC_50_) and (LC_90_) values were calculated using the non-linear regression function of GraphPad Prism and compared using 1-way ANOVA with Tukey's multiple comparisons post-test.

## Results

### Fungal susceptibility

**Figure 2:**
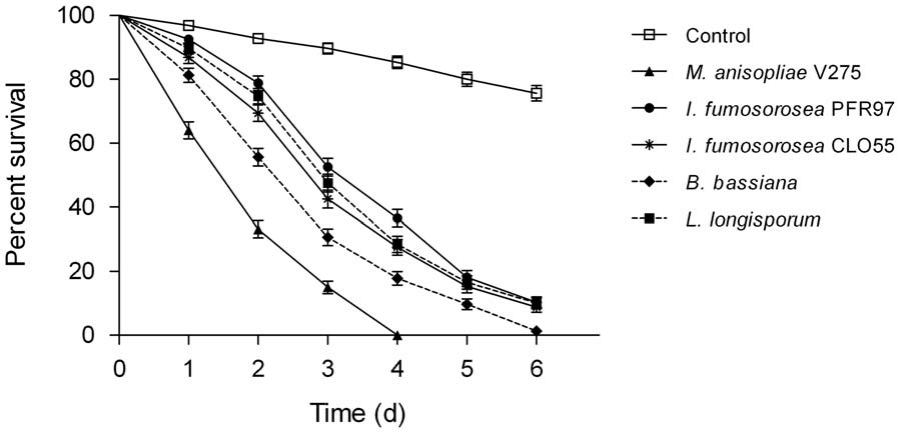
Effect of fungal infection on midge survival. Mean (± SEM) cumulative proportional survival of adult *Culiocides nubeculosus* exposed for 6 days to ‘dry’ conidia of entomopathogenic fungi *Metarhizium anisopliae* V275, *Isaria fumosorosea* PFR97, *Isaria fumosorosea* CLO55, *Beauveria bassiana*, *Lecanicillium lecanii* (10^11^ conidia m^−2^ on tissue paper) and uninfected control at 20±1°C. Controls were not exposed to any fungus (‘0’ dose). Data represent survival of eight replicates of approximately 40 adult males and females/replicates.

All fungal isolates significant reduced midge survival compared with untreated controls 6 days after exposure (*P*<0.001, Kaplan-Meier log-rank pairwise comparison, Fig. 2). Overall, *M. anisopliae* V275 was the most effective fungus and caused a significantly greater reduction in midge survival compared with all other fungus species (*P*<0.001, Kaplan-Meier log-rank pairwise comparison). Following continuous exposure, 100% mortality (confirmed by fungal sporulation on midge cadavers) was observed with *M. anisopliae* V275 by day 4 compared to estimated cumulative mortalities of 80.2±2.1% 72.5±2.5%, 71.6±2.5% and 63.4±2.7% for *B. bassiana*, *P. fumosorosea* CLO55, *L. longisporum* and *P. fumosorosea* PRF97, respectively. Control treatments showed 24.4±2.4% (with 0% sporulation) midge mortality 6 days after treatment.

The LT_50_ and LT_90_ values for midges exposed to different fungus species/strains differed significantly (*P*<0.001). The lowest LT_50_ and LT_90_ were from *M. anisopliae* V275, whereas the highest LT_50_ and LT_90_ values were from *P. fumosorosea* PRF97 (Table 1).

### Dose and exposure responses

**Figure 3:**
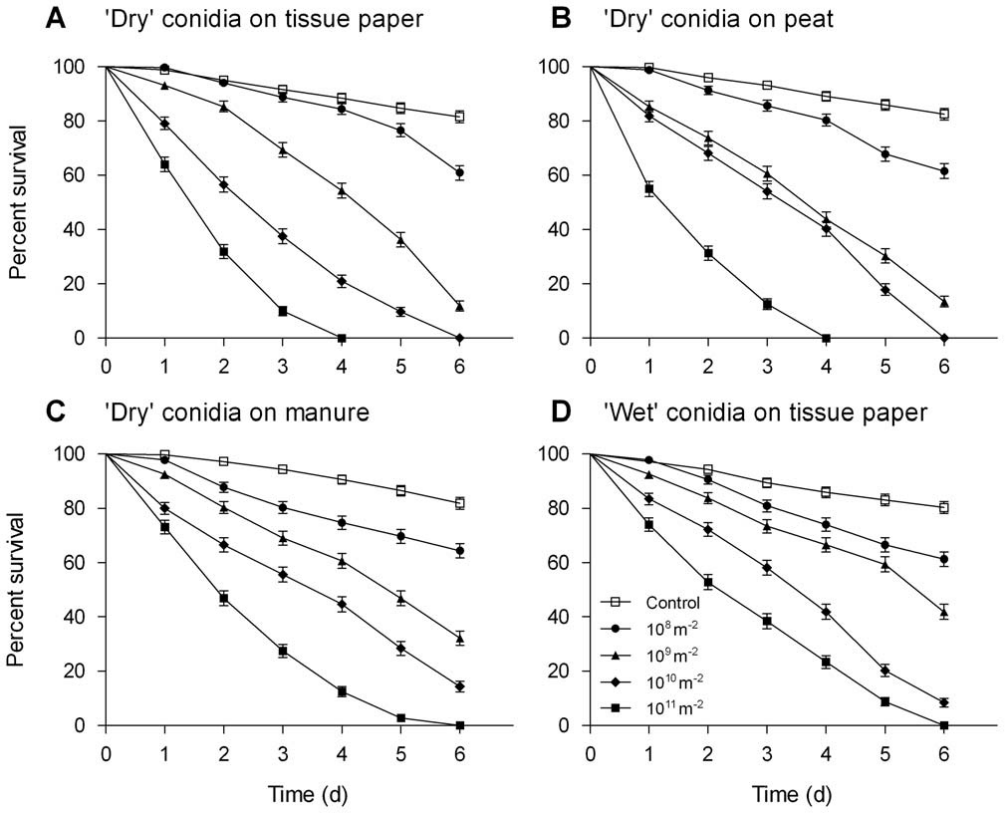
Influence of substrates. Survival curves (Mean±SEM) for adult *Culiocides nubeculosus* exposed for 6 days to different doses (10^8^–10^11^ conidia m^−2^) of ‘dry’ and ‘wet’ conidia of *Metarhizium anisopliae* V275 on separate substrates at 20±1°C. Controls were not exposed to any fungus (‘0’ dose). Data represent survival of eight replicates of approximately 40 adult males and females/replicate.

**Figure 4:**
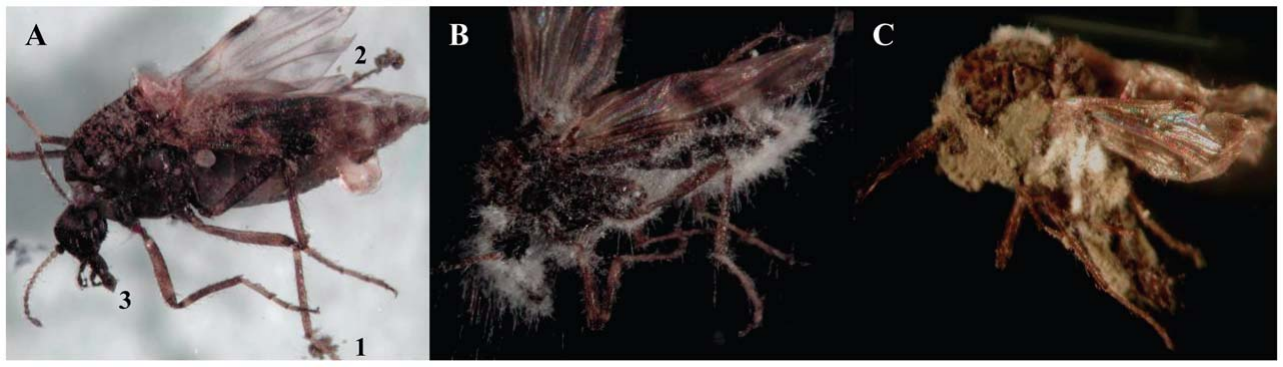
*Culiocides nubeculosus* midges at different times after contact with ‘dry’ conidia of *Metarhizium anisopliae* V275. (A) Adult *Culiocides nubeculosus* 1 day after death showing conidial attachment to the ‘feather’ of the last few tarsae (1), and several on the ‘feather’ of the tibia (2) and around the proboscis (3). (B) An adult midge cadaver 3 days after death showing hyphal growth of *Metarhizium anisopliae* V275. (C) An adult midge cadaver 6 days after death showing sporulation of fungus.

*M. anisopliae* caused a significant reduction in midge survival at all doses tested (10^8^–10^11^ conidia m^−2^) and on all substrates (‘dry’ conidia on tissue paper, peat and horse manure and ‘wet’ conidia on tissue paper) compared with untreated controls (*P*<0.001, Kaplan-Meier log-rank pairwise comparison, Fig. 3). In all case where midges were exposed to ‘dry’ conidia, fungal sporulation was observed. In dose response tests the lowest dose resulting in a significant effect on midge survival was 10^9^ conidia m^−2^ (10^5^ conidia cm^−2^; Fig. 3). Many conidia were found attached to the ‘feathers’ of the last tarsae, and also frequently on the ‘feathers’ of the tibia and around the proboscis (Fig. 4A–B).

There were no significant differences (*P*>0.05) observed between LC_50_ and LC_90_ values when comparing ‘dry’ conidia applied to tissue paper or peat (Table 2; 1-way ANOVA, Tukey's multiple comparison post-test) although both LC_50_ values were significantly lower than those for ‘dry’ conidia applied to horse manure or ‘wet’ conidia on tissue paper (Table 2; *P*<0.001, 1-way ANOVA, Tukey's post-test).

**Table 2:** Substrate influence median lethal dose.

Fungal application method/substrates	LC_50_ m^−2^ *	LC_90_ m^−2^ *
‘Dry’ conidia on tissue paper	2.4 (2.0–2.8)×10^7^	1.5 (1.1–2.0)×10^8^
‘Dry’ conidia on peat	2.5 (2.1–3.0)×10^7^	1.7 (1.2–2.3)×10^8^
‘Dry’ conidia on manure	6.0 (3.6–10.0)×10^7^	2.1 (0.5–8.2)×10^9^
‘Wet’ conidia on tissue paper	9.0 (5.7–14.1)×10^7^	4.5 (1.2–16.7)×10^9^

Adult midges were exposed to substrates treated with different doses (0, 10^8^, 10^9^, 10^10^ and 10^11^ m^−2^) of ‘dry’ and ‘wet’ conidia of *Metarhizium anisopliae* V275 at 20±1°C. Controls were not exposed to any fungus (‘0’ doses). Dead midges were collected daily from each container for 6 days and kept at 25°C for sporulation.

* =  Mean lethal dose estimated from five doses (four replicates/dose; approximately 40 adult males and females/replicate). 95% Confidence intervals in parentheses.

**Figure 5:**
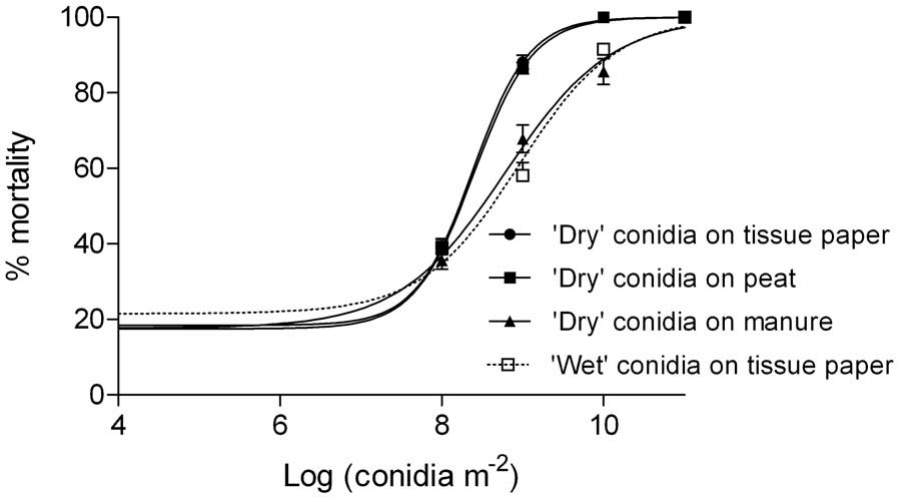
Effect of substrates on fungus dose. Mean (± SEM) mortality of adult *Culiocides nubeculosus* 3–4 days of age, 6 days post exposure to different doses (10^8^–10^11^ conidia m^−2^) of ‘dry’ and ‘wet’ conidia of *Metarhizium anisopliae* V275 on separate substrates at 20±1°C. Controls were not exposed to any fungus (‘0’ doses). The sigmoidal models were fitted to the data using nonlinear regression. Data represent survival of eight replicates of approximately 40 adult males and females/replicate.

Midge survival data from all the substrates fitted closely to Gompertz distribution models (Fig. 5). Estimates of daily survival rates derived from the Gompertz model [19] showed a dramatic reduction following exposure to conidia (Fig. 5). In dose-response experiments, daily survival rates were inversely related to the exposure dose. Figure 5 shows the survival curves after 6 days for adult midges exposed to different doses of conidia.

### Influence of substrate and formulation

**Figure 6:**
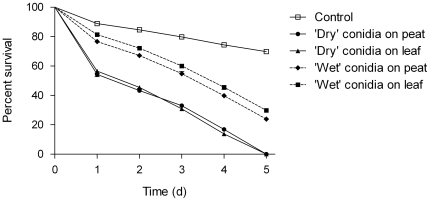
Effects of substrate and formulation. Mean (± SEM) cumulative proportional survival of adult *Culiocides nubeculosus* exposed for 6 days to ‘dry’ and ‘wet’ conidia of *Metarhizium anisopliae* V275 in cages within a greenhouse. Treatments consisted of ‘dry’ or ‘wet’ conidia at dose of 2.5×10^9^ conidia m^−2^ dusted or applied on peat and leaf litter. Corresponding control groups were exposed to the solvent only. Data represent survival of six replicates of approximately 200 adult males and females/replicate.

All applications of *M. anisopliae* significantly reduced midge survival compared to controls (*P*<0.001, Kaplan-Meier log-rank pairwise comparison, Fig. 6). There was no significant difference between substrates for ‘dry’ conidia (Kaplan-Meier log-rank pairwise comparison) with 100% mortality (confirmed by fungal sporulation on midge cadavers) observed after 5 days on both peat and leaf litter. Applications of ‘dry’ conidia caused significantly greater mortality than ‘wet’ conidia on the same substrate (*P*<0.001; Kaplan-Meier log-rank pairwise comparison). No fungal sporulation was observed in control midges.

All surviving midges from fungal-treated substrates were found to be infected by *M. anisopliae* V275 and subsequently developed a covering of conidiophores and conidia (Fig. 4C).

## Discussion

This is the first study to demonstrate the efficacy of an entomopathogenic fungus against adult *C. nubeculosus*. Whilst all fungal species tested significantly reduced midge survival, strain V275 of *M. anisopliae* was the most infective and virulent. Any reduction in midge survival will likely reduce the number of blood meals taken, and therefore the likelihood of the vector acquiring and transmitting a pathogen. Indeed, previous studies have demonstrated that infection of adult mosquitoes (*Anopheles gambiae*, *Culex quinquefasciatus*, *Aedes aegypti* and *A. albopictus*) with *M. anisopliae* causes a significant reduction in their survival and disease transmission under field conditions [20], [21], [11]. Reducing adult survival is therefore considered the most effective way to reduce disease transmission.

*M. anisopliae* showed a clear dose-dependent effect on mortality in adult *C. nubeculosus* on all substrates. Both the speed of kill and the number of midges showing infection after death increased with increasing fungal dose applied. Application of ‘dry’ conidia to the surface of tissue paper or peat had a greater effect than ‘wet’ conidia. Higher LC_50_ and LC_90_ values were also observed when ‘dry’ conidia were applied to manure rather than peat or tissue paper, suggesting that virulence was influenced by both the substrates and the formulation (‘dry’ versus ‘wet’ conidia). The dose response tests showed that midges can pick up a lethal dose of fungal conidia from different substrates within a short period of time (i.e mortality was evident after 24 hrs), confirmed by microscopic observation of dead midges. Many conidia were found attached to the last tarsae and several at the tibia and around the proboscis. While the effective conidial dose (i.e. conidia that actually attach to the midge's cuticle and subsequently invade the integument and haemocoel) is unknown, it is likely that it is a rather low proportion of the conidia that attach.

The results from the laboratory trials are supported by the subsequent greenhouse study that also found ‘dry’ conidia considerable outperformed ‘wet’ conidia. ‘Dry’ conidia of *M. anisopliae* have been shown to be very effective in infecting mosquitoes [22], [23] although the greenhouse trials in this study used a conidial dose (2.5×10^9^ m^−2^) almost 10-fold lower than that used against mosquitoes (2×10^10^ m^−2^). Previous workers have also found that ‘dry’ conidia kill mosquitoes faster than oil formulated ones [23] and it is possible that adhesive factors are removed by the carrier. In addition, ’wet’ conidia are much less likely to attach to adult midges in natural conditions and will quickly settle out onto less accessible surfaces [24], resulting in a substantial loss of conidia through sinking (>90%) [25], whereas ‘dry’ conidia do not sink after application [26]. It should be noted however, that field applications of ‘dry’ conidia may lose their virulence within days because of environmental conditions (notably UV radiation, humidity and high temperature).

Whatever the application method or substrates used, all surviving adults taken from *M. anisopliae*-treated substrates in the greenhouse study subsequently proved to be infected with the pathogen. This observation suggests these adults were at the early stage of fungal infection when trapped. It is therefore possible that conidial transmission between adult midges (especially between males and females) in the field may cause further infections within the population. Horizontal transfer for *M. anisopliae* has been demonstrated from honeybees to the pollen beetle *Meligethes aeneus* [27] and between mosquitoes *A. gambiae* [28]. The conditions under which conidial transmission is likely to occur are quite specific however, and field verification is required to measure its real impact.

Overall, our results suggest that entomopathogenic fungi present a potential method for targeting adult biting midges and the arboviruses they transmit as part of a wider integrated programme. The most effective strain in this study (*M. anisopliae* strain V275) is commercially available (F52, Novozymes, USA) and the production and application of fungi both involve relatively simple infrastructures and processes. This fungus therefore has the potential to be a cost effective and relatively straightforward weapon against arboviruses. However, feasibility and sustainability of the use of fungi as a vector control method in the field will depend upon the choice of fungal isolate and formulation. The choice of application and delivery methods will highly influence the infection coverage and the effectiveness of fungi in the field. Field experiments will also need to thoroughly investigate potential effects on non-target species, although other workers have already found that *M. anisopliae* is safe for birds, fish and mammals [29], [30] and poses no obvious risk to humans or the environment [31], [32], [33]. There remains a need to test the fungus in large-scale field trials with the eventual aim of developing protocols for its simply and economical application in BT endemic developing countries.
